# Spontaneous Spinal Cord Infarction Presenting as Anterior Spinal Artery Syndrome: A Case Report

**DOI:** 10.7759/cureus.116043

**Published:** 2026-09-10

**Authors:** Daniela Soares, Pedro Tavares Almeida, Henrique Costa, Pedro Barros, Tiago Gregório

**Affiliations:** 1 Internal Medicine, Local Health Unit of Entre Douro e Vouga, Santa Maria da Feira, PRT; 2 Neurology, Unidade Local de Saúde de Gaia e Espinho, Vila Nova de Gaia, PRT; 3 Internal Medicine, Unidade Local de Saúde de Gaia e Espinho, Vila Nova de Gaia, PRT

**Keywords:** acute myelopathy, anterior spinal artery syndrome, magnetic resonance imaging, paraparesis, spinal cord infarction

## Abstract

Spinal cord infarction is a rare cause of acute myelopathy and may be difficult to recognize when it occurs outside a traumatic or perioperative setting. We report a 68-year-old man with hypertension, type 2 diabetes mellitus, dyslipidemia, and active smoking who presented with sudden severe low back pain followed within one hour by rapidly progressive bilateral lower-limb weakness and sensory loss. Neurologic examination showed severe paraparesis, absent lower-limb reflexes, impaired pain sensation below T10, and relative preservation of proprioception. Aortic computed tomography angiography excluded dissection, and spinal magnetic resonance imaging demonstrated T2 hyperintensity with diffusion restriction in the anterior gray matter from T10-T11 to the conus medullaris, consistent with acute infarction in the anterior spinal artery territory. The patient received antiplatelet therapy, high-intensity statin therapy, venous thromboembolism prophylaxis, bladder management, and early rehabilitation. At follow-up, left lower-limb strength improved to 4/5, while right lower-limb plegia, neurogenic bladder, and wheelchair dependence persisted. This case emphasizes the importance of recognizing the characteristic clinical pattern of anterior spinal artery syndrome, urgently excluding aortic and compressive causes, obtaining diffusion-weighted spinal imaging, and initiating multidisciplinary supportive and rehabilitative care.

## Introduction

Spinal cord infarction is an uncommon vascular cause of acute myelopathy. A population-based study reported an age- and sex-adjusted incidence of 3.1 cases per 100,000 person-years [1]. Although spinal cord ischemia is classically associated with aortic disease, aortic surgery, systemic hypoperfusion, or other procedural settings, spontaneous non-traumatic events also occur and can be diagnostically challenging [2-4]. The anterior spinal artery supplies approximately the anterior two-thirds of the spinal cord, and infarction in this territory typically produces abrupt motor dysfunction, autonomic involvement, and loss of pain and temperature sensation, with relative preservation of dorsal-column modalities [2,3].

Magnetic resonance imaging (MRI), particularly when diffusion-weighted imaging (DWI) and apparent diffusion coefficient (ADC) maps are included, is central to confirming the diagnosis and excluding compressive or inflammatory mimics [2,3]. Because randomized treatment trials and disease-specific guidelines are lacking, acute management is individualized and generally focuses on identifying reversible causes, treating vascular risk factors and complications, and initiating rehabilitation [4]. We present a spontaneous spinal cord infarction with a clinical and radiologic anterior spinal artery syndrome to highlight the diagnostic reasoning, management limitations, and functional evolution of this rare neurologic emergency.

## Case presentation

A 68-year-old man with poorly controlled hypertension, type 2 diabetes mellitus, and dyslipidemia, as well as active tobacco use, presented to the emergency department with sudden-onset severe low back pain followed by progressive loss of strength and sensation in both lower limbs. Symptoms had begun approximately one hour before presentation. He denied trauma, previous spinal or aortic surgery, chest pain, interscapular pain, fever, or recent infection. He initially denied sphincter symptoms.

On arrival, he was hemodynamically stable, alert, cooperative, and fully oriented. There were no abnormalities of higher cortical function or cranial nerves. Neurologic examination showed severe bilateral lower-limb weakness (Medical Research Council grade 1/5), absent lower-limb deep tendon reflexes, and bilateral indifferent plantar responses. Pinprick sensation was reduced below a T10 sensory level, whereas proprioception was relatively preserved, suggesting an acute anterior spinal cord syndrome.

Initial laboratory evaluation, including complete blood count, routine biochemistry, thyroid function, coagulation studies, and an extended autoimmune and infectious workup, did not reveal a clinically relevant abnormality. Serologic testing for hepatitis B, hepatitis C, human immunodeficiency virus, and syphilis was negative. Lumbar computed tomography did not identify a structural cause for the deficit. Computed tomography angiography of the aorta excluded dissection and other acute aortic pathology, showing only small calcified parietal atherosclerotic plaques.

Spinal MRI, performed approximately 18 hours after symptom onset, demonstrated T2 hyperintensity and restricted diffusion involving the anterior horns of the spinal gray matter from T10-T11 to the conus medullaris at L1-L2, with corresponding low signal on the ADC map. These findings were consistent with acute spinal cord infarction in the anterior spinal artery territory (Figure 1). No adjacent vertebral body signal abnormality or traumatic lesion was identified.

**Figure 1:**
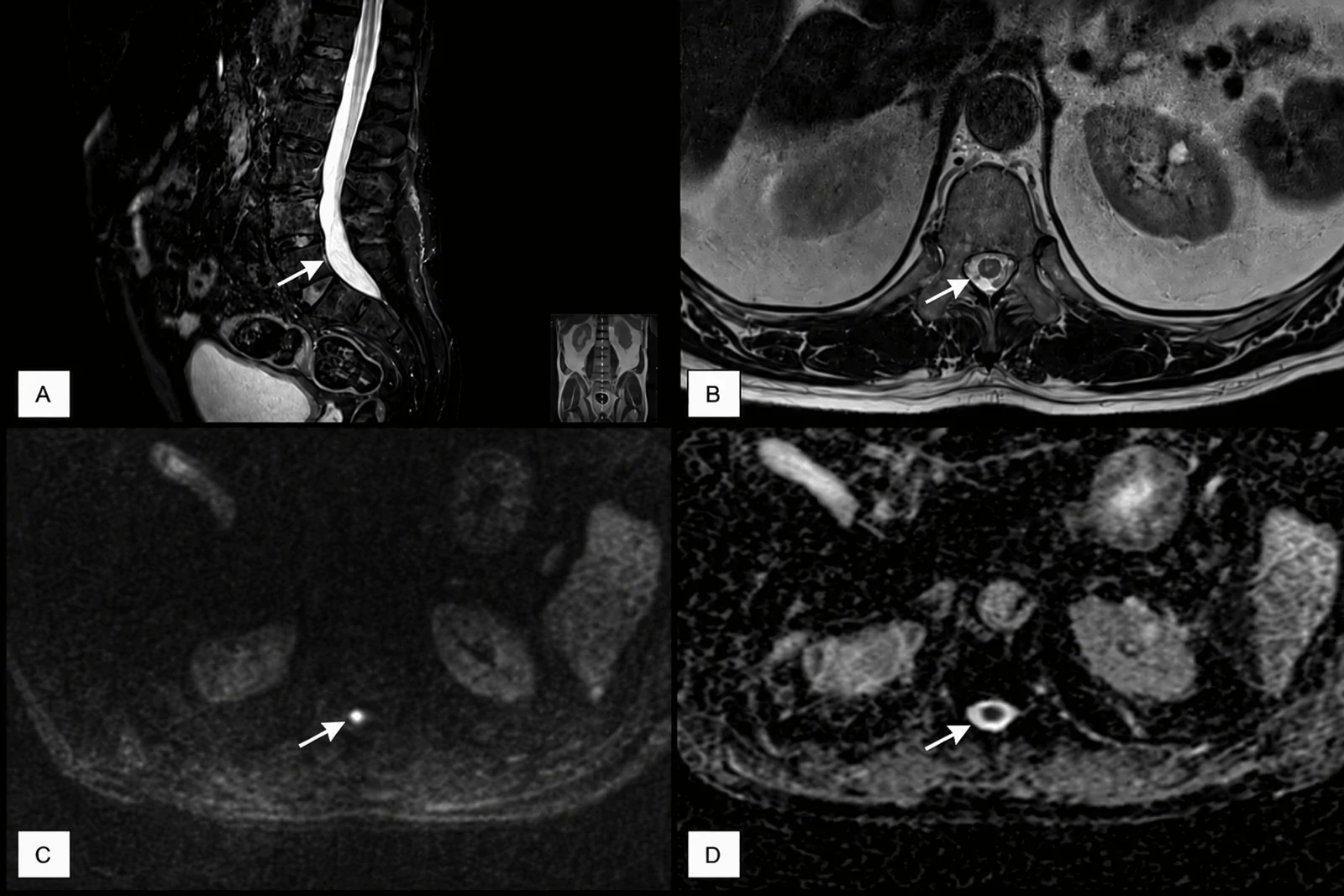
Thoracolumbar spinal magnetic resonance imaging demonstrating acute spinal cord infarction. (A) Sagittal T2/STIR-weighted image showing intramedullary hyperintensity at the thoracolumbar level. (B) Axial T2-weighted image demonstrating hyperintensity involving the anterior horns of the spinal gray matter. (C) Axial diffusion-weighted imaging (DWI) showing hyperintensity in the affected anterior spinal cord. (D) Corresponding apparent diffusion coefficient (ADC) map showing low signal, confirming restricted diffusion. MRI: magnetic resonance imaging; DWI: diffusion-weighted imaging; ADC: apparent diffusion coefficient

Because diagnostic confirmation was obtained approximately 18 hours after symptom onset, systemic thrombolysis was not performed, as this was beyond the conventional therapeutic window extrapolated from cerebral ischemic stroke and used by the treating team. The patient was started on acetylsalicylic acid 100 mg/day and high-intensity atorvastatin 40 mg/day, together with prophylactic enoxaparin. Acute urinary retention subsequently required bladder catheterization.

As part of the etiologic evaluation, a 12-lead electrocardiogram showed sinus rhythm at 67 beats/minute, and transthoracic echocardiography revealed no relevant abnormalities. An extended thrombophilia evaluation did not identify an established prothrombotic disorder. The laboratory report included heterozygous MTHFR c.677C>T and PAI-1 -675 4G variants; these findings were not considered sufficient to establish the mechanism of the infarction.

Rehabilitation was initiated during the hospitalization, with progressive transfer to a chair and structured mobilization. Despite the severe paraparesis documented at admission (MRC grade 1/5 bilaterally), motor function continued to deteriorate, and by hospital day five both lower limbs were plegic, although there was mild improvement in left-sided sensory function. The patient was transferred to a neurologic rehabilitation center and later continued a specialized rehabilitation program after hospital discharge. At one-year follow-up, left lower-limb strength had improved to 4/5, while right lower-limb plegia persisted. He continued to have a dorsal sensory level and neurogenic bladder and remained unable to walk independently, requiring a wheelchair for mobility. Despite these limitations, he retained independence in his other activities of daily living.

## Discussion

This case illustrates a classic clinical-radiologic pattern of spontaneous spinal cord infarction affecting the anterior spinal artery territory. The abrupt onset, severe low back pain, rapid progression to marked paraparesis, selective impairment of pain sensation with relative preservation of proprioception, and DWI/ADC restriction strongly supported a vascular myelopathy [2,3]. In the emergency setting, the principal alternatives include compressive myelopathy, transverse myelitis, spinal epidural hematoma, fibrocartilaginous embolism, and, in selected presentations, peripheral disorders such as Guillain-Barré syndrome. The combination of a distinct sensory level and an anterior cord imaging pattern was particularly helpful in localizing the lesion to the spinal cord.

Urgent exclusion of aortic disease is critical because aortic dissection and other aortic pathologies can interrupt radiculomedullary perfusion and require a fundamentally different therapeutic approach [3,4]. In this patient, aortic computed tomography angiography showed no dissection or aneurysmal complication. No inflammatory, infectious, compressive, or traumatic cause was identified. Given the marked burden of vascular risk factors and the presence of aortic atherosclerotic plaque, an atherosclerotic or atheroembolic mechanism was considered plausible, although no culprit vessel occlusion was demonstrated and the precise mechanism therefore remains presumptive.

The genetic findings deserve cautious interpretation. Heterozygous MTHFR c.677C>T is common and should not be classified as an inherited thrombophilia or used to infer an increased risk of arterial or venous thrombosis [5]. Accordingly, the MTHFR result was not used to assign causality. The PAI-1 4G finding was likewise treated as non-diagnostic in the absence of an established prothrombotic phenotype. This distinction is important because over-attribution of common polymorphisms can obscure more plausible vascular mechanisms and may lead to unnecessary long-term antithrombotic strategies.

There is no established reperfusion standard for spontaneous spinal cord infarction. Most recommendations are extrapolated from cerebral ischemic stroke or derived from case reports and small observational series [4,6]. Intravenous thrombolysis has been reported with alteplase and tenecteplase, including treatment beyond 4.5 hours in selected cases, but the available evidence is insufficient to establish efficacy, optimal patient selection, or a validated therapeutic window [7,8]. A nationwide observational analysis also found that thrombolysis was rarely used and did not demonstrate clear differences in major in-hospital outcomes, although residual confounding and coding limitations restrict causal interpretation [9]. In the present case, thrombolysis was not administered because diagnostic confirmation was obtained approximately 18 hours after symptom onset, beyond the conventional cerebral ischemic stroke thrombolysis window used by the treating team. Antiplatelet therapy and high-intensity statin therapy were therefore selected in the context of a presumed atherosclerotic mechanism and high vascular risk.

Functional outcome after spinal cord infarction is heterogeneous and is strongly influenced by the severity of the neurologic deficit at nadir. In a long-term cohort, substantial recovery continued after hospital discharge, and some patients who initially required a wheelchair later regained ambulatory ability [10]. More recent systematic data similarly suggest that neurologic improvement is possible in a meaningful proportion of patients with spontaneous spinal cord infarction [4]. In our patient, recovery was asymmetric: left lower-limb power improved to near-antigravity functional strength, while the right lower limb remained plegic and bladder dysfunction persisted. This pattern illustrates both the potential for delayed recovery and the substantial long-term disability that can follow a severe initial infarction. Early multidisciplinary rehabilitation remains a central component of supportive care, even though high-quality evidence defining its optimal timing and intensity specifically for spinal cord infarction is limited.

The principal limitation of this report is the absence of a definitively demonstrated vascular culprit. Although the clinical presentation and MRI findings satisfy strong diagnostic features of spinal cord infarction, the presumed atherosclerotic mechanism remains an inference. The case nevertheless provides a practical emergency-department lesson: a patient may arrive early after symptom onset, but the rarity of spinal cord infarction and the time required to exclude mimics can delay diagnostic confirmation and narrow potential reperfusion options.

## Conclusions

Spinal cord infarction should be considered in any patient with abrupt non-traumatic paraparesis or paraplegia, particularly when a sensory level and dissociated sensory loss suggest an anterior spinal cord syndrome. Rapid spinal MRI with diffusion-weighted sequences and urgent exclusion of aortic and compressive pathology are essential to establish the diagnosis and identify treatable alternatives. Because disease-specific acute treatment evidence remains limited, management should be individualized and should include prevention of secondary complications, aggressive control of vascular risk factors when appropriate, and early multidisciplinary rehabilitation. Long-term recovery can be clinically meaningful but incomplete, making careful prognostic counseling and sustained rehabilitation important components of care.
